# Management of COVID-19 Response in a Secure Forensic Mental Health Setting: Réponse à la gestion de la COVID-19 dans un établissement sécurisé de santé mentale et de psychiatrie légale

**DOI:** 10.1177/0706743720935648

**Published:** 2020-06-23

**Authors:** Alexander I. F. Simpson, Sumeeta Chatterjee, Padraig Darby, Roland M. Jones, Margaret Maheandiran, Stephanie R. Penney, Tania Saccoccio, Vicky Stergiopoulos, Treena Wilkie

**Affiliations:** 1 Complex Care and Recovery Program, Centre for Addition and Mental Health, Division of Forensic Psychiatry, Department of Psychiatry, University of Toronto, Ontario, Canada

**Keywords:** COVID-19, secure hospital, forensic mental health, isolation units

## Abstract

**Objectives:**

The coronavirus disease 2019 (COVID-19) pandemic presents major challenges to places of detention, including secure forensic hospitals. International guidance presents a range of approaches to assist in decreasing the risk of COVID-19 outbreaks as well as responses to manage outbreaks of infection should they occur.

**Methods:**

We conducted a literature search on pandemic or outbreak management in forensic mental health settings, including gray literature sources, from 2000 to April 2020. We describe the evolution of a COVID-19 outbreak in our own facility, and the design, and staffing of a forensic isolation unit.

**Results:**

We found a range of useful guidance but no published experience of implementing these approaches. We experienced outbreaks of COVID-19 on two secure forensic units with 13 patients and 10 staff becoming positive. One patient died. The outbreaks lasted for 41 days on each unit from declaration to resolution. We describe the approaches taken to reduction of infection risk, social distancing and changes to the care delivery model.

**Conclusions:**

Forensic secure settings present major challenges as some proposals for pandemic management such as decarceration or early release are not possible, and facilities may present challenges to achieve sustained social distancing. Assertive testing, cohorting, and isolation units are appropriate responses to these challenges.

## Abstract (fr)

**Objectifs:**

La pandémie de la COVID-19 présente des difficultés majeures aux établissements de détention, y compris les hôpitaux de psychiatrie légale sécurisés. Les guides internationaux suggèrent une série d’approches qui aident à diminuer le risque d’épidémies de COVID-19 et qui proposent des réponses pour prendre en charge les éclosions d’infection, le cas échéant.

**Méthodes:**

Nous avons mené une recherche dans la littérature sur la prise en charge de la pandémie ou d’épidémies dans les établissements de santé mentale et de psychiatrie légale, notamment dans des sources de littérature grise, de 2000 à avril 2020.

**Résultats:**

Nous avons trouvé une gamme de guides utiles, mais aucune expérience publiée de la mise en œuvre de ces approches. Nous décrivons les approches employées pour réduire le risque d’infection, la distanciation sociale et les changements du modèle de prestation des soins. Nous décrivons l’évolution de l’épidémie de COVID-19 dans votre propre établissement, ainsi que la conception et les effectifs requis d’une installation d’isolement légiste.

**Conclusions:**

Les établissements de psychiatrie légale présentent des difficultés majeures car certaines propositions de gestion de la pandémie, comme la décarcération ou la libération précoce ne sont pas possibles, et les établissements peuvent poser des difficultés pour atteindre une distanciation sociale soutenue. Un dépistage ferme, le regroupement et les installations d’isolement sont des réponses appropriées à ces difficultés.

## Introduction

The coronavirus disease 2019 (COVID-19) pandemic has presented huge challenges to health services. Serious concern has arisen regarding how to protect persons in places of confinement such as long-term care facilities and places of detention such as jails, prisons, and secure forensic services,^1^ with particular concerns that arise for persons with serious mental illness.^2^ Rapid spread of COVID-19 has resulted in tragic circumstances in long-term care facilities in multiple countries. In Wuhan, China, one facility suffered multiple fatalities due to isolation of large patient numbers and staff in circumstances of poor infection control.^3^ A serious outbreak in a South Korean psychiatric facility resulted in 7 deaths.^4^ There have not been any documented outbreaks of COVID-19 in forensic mental health services internationally. Long-term care facilities for the elderly have tragically been a major center of mortality.^5^

Secure (forensic) mental health hospitals present a unique challenge. First, residents of such facilities have major mental illnesses often with significant physical comorbidity making them a high-risk population to adverse outcomes from COVID-19. Second, secure hospitals are spaces of communal living, with many residents required to share facilities and participate in group therapies in close proximity to one another. Third, the long-term hospitalization of forensic patients is often a result of serious risk to their own health and safety or that of others. Commonly, patients experience difficulties with emotional regulation and behavioral controls, resulting in difficulties in following universal instructions for infection control such as maintaining physical distance, hand hygiene, or wearing surgical masks. Some of these issues may overlap with the contained, vulnerable patient populations in long-term care facilities; however, an important point of difference is that patients in the care of long-term care facilities often have limited mobility, while forensic patients are usually mobile, and sometimes behaviorally disturbed, requiring physical intervention from staff to manage their risk of harm to themselves or others.

Some inpatient and criminal justice settings have worked to release residents who are able to return the community; discharging forensic patients rapidly is usually not feasible. Policy changes can help isolate forensic populations from the community spread of infection but the ability to maintain effective social distancing within the institution may be limited, reducing the ability to control the spread of COVID-19 within such settings. This is strongly influenced by architectural factors as well as patient and staff factors.

We searched MEDLINE, Embase, and PubMed for any published articles relating to COVID in forensic, secure, or state psychiatric settings from 2000 to April 30, 2020. Our search was expanded to the gray literature where we utilized the same criteria and applied those to a Google hand search. The search was focused on industry-relevant websites such as the WHO, NHS, CDC, APA, and SAMHSA. We found no published literature on modeling the dynamics of COVID-19 spread or responses in forensic secure hospitals.

The recommendations of major international organizations including WHO,^6^ CDC,^7^ and the European CDC^8^ regarding the management of pandemic events including in hospital settings and places of detention are clearly well informed by infection control principles and relevant to secure hospitals. As of yet, however, there is no empirical evidence on the application of these principles to secure hospital settings. The purpose of this article is to describe the approach taken by a 182-bed forensic program within a large psychiatric facility to plan for and manage the early phase of the COVID-19 outbreak.

## The Setting

The Centre for Addiction and Mental Health (CAMH) is a 530-bed psychiatric hospital in Toronto, ON, Canada. It includes the largest forensic program in Ontario, which serves the Greater Toronto Area with a population of approximately 3.0 million people. The forensic program encompasses 182 medium and minimum secure forensic beds across 8 units. These units are about 50 years old and cramped with individual bedrooms but shared lounge, dining, and bathroom facilities. There are both single and mixed gender units. Units are highly staffed. Patients to staff member ratios over a 24-hr period vary from 1:1.1 (1.1 staff for every patient over the 24-hr period in low secure) to 1:2.3 (acute medium secure units). Staff accommodation is also cramped. The majority of patients have extensive off unit passes, including unaccompanied passes for therapeutic and recreational purposes.^9^

## The Patient Population

Forensic patients are those found to be either unfit to stand trial (UST) or not criminally responsible on account of mental disorder (NCRMD) and dispositions of such persons (approximately 4,600 individuals in Canada) are overseen by provincial review boards (in Ontario, the Ontario Review Board [ORB]).

All are over the age of 18, fewer than 10% of patients are over 65 years of age.^10,11^ About 90% have been found NCRMD, the remainder are UST with about 15% women. Upward of 80% have a primary psychotic illness, another 10% an affective psychosis, and about 10% a primary developmental disorder. Comorbid substance abuse problems affect approximately 50% of patients. Violent index offenses are the majority, about 15% homicide or attempted homicide, and about 10% to 15% have an index sexual offense. Mean age at entering the forensic system is in the mid 30s, and median time to absolute discharge was 7 to 8 years. The ethnic makeup of forensic patients is becoming more diverse, with half of the forensic patients being Canadian born, the remainder being voluntary (40%) or forced (10%) migrants.

## Patient Pathways

The forensic program at CAMH admits about 150 patients per year. The majority of those are persons referred by the courts for restoration of fitness to stand trial or persons found NCRMD and receiving a hospital disposition from the ORB. About a third of admissions are of forensic community patients requiring readmission for a variety of reasons, usually medication noncompliance, symptom exacerbation, or reemergence or substance use.

## Challenge of COVID-19

Like all major institutions, CAMH undertakes extensive pandemic planning applying lessons learned from the SARS epidemic of 2003. Many of the initiatives undertaken in the forensic program were institution-wide policies and approaches adapted for the forensic setting. As the risk of the pandemic emerged through January and February 2020, this plan was enacted and early stages of planning commenced for the implementation of graded responses to the pandemic, aligned with another major health institutions and public health recommendations. The way in which these policies were applied to the forensic program is described below.

### General Infection Control Approaches

*Education:* The principle approach by interprofessional teams was to provide education and support to patients about the risks that COVID-19 posed and the rationale for the precautions as set out below.
*Basic hygiene:* Hand hygiene and the availability of hand cleanser was emphasized to the inpatient units with extra effort to educate patients and staff about the necessity of appropriate hand hygiene and the provision of hand sanitizers. Instruction about how to cough safely and dispose of tissues was explained extensively.
*Disinfection:* Increased attention was paid to cleaning but particularly to surfaces where droplet-based spread might be of particular relevance.
*Physical distancing:* Adaptation of secure units to accommodate physical distancing commenced, including redesign of work plans and workstations to spread staff out as much as possible within previously cramped areas. Communal dining was replaced by patients either eating in their own rooms or having 2 to 3 waves of serving meals with adequate physical distancing being required in the dining room. Physical distancing guidelines were issued for elevators as well as 2-m markers in high traffic areas.
*Screening of all persons entering the facility:* Prominent risk on inpatient units was of visitors or staff bringing virus into inpatient settings. Screening of all persons entering inpatient units was commenced on March 16 including screening for travel, respiratory symptoms, and contact with anyone who is in high-risk situations. Every staff member was required to go through screening daily.
*Restriction of visits/students:* Visits were initially reduced and then placed on a virtual or phone basis only from March 12. Students and trainees were removed from clinical areas early in the pandemic.
*Personal Protective Equipment (PPE):* The use of PPE was controversial. Along with screening, the requirement for staff to wear a surgical mask in all clinical areas and during all interactions with other staff members was introduced on March 27. On outbreak units (or suspected outbreak units), the use of full PPE including procedure masks, gowns, gloves, goggles/masks was introduced for staff. Patients were not provided with face masks but expected to observe droplet and contact precautions.
*Cessation of unescorted and community passes:* In keeping with the community principle of isolation that was part of the provincial state of emergency in Ontario, we initially reduced and then ceased all unaccompanied passes into the community or on the hospital grounds. We were aware that although the hospital is smoke-free, patients on unaccompanied passes frequently congregated in park areas and shared cigarettes and lighters. We were concerned that this would expose patients to a high risk of acquiring COVID-19 and ceased all of these passes on March 23. This also had the effect of reducing access to some community-based rehabilitation opportunities.

### Changes to Care Delivery

*Reduction in group-based programs:* Aligned with social distancing principles, if group-based interventions could not occur with adequate physical distancing they were ceased on March 11 or made virtual. There was considerable innovation of allied health team members to support on-unit programming, small-group walks for fresh air, individual treatment in place of group and virtual group modalities.
*Rotation of on-unit psychiatrists with virtual team meetings:* All inpatient units have at least two attending psychiatrists. In order to preserve the psychiatric workforce and reduce unnecessary risks of infection on inpatient units, we commenced the process by which psychiatrists would alternate the weeks in which they were in physical attendance on the units. All teams were consulted about this and all agreed this was workable and appropriate. To facilitate ongoing engagement of both psychiatrists with the interdisciplinary team and patients, virtual care was introduced. This involved the adoption of secure web-based conferencing (Webex) for team meetings, morning huddles, and patient interviews. The on-unit psychiatrist could assess any patient unable to use a virtual format and most psychiatrists’ weekly patient reviews were performed by the usual attending psychiatrist either on unit or remotely. This process was rapidly adopted and appreciated by both patients and staff. The patient community repeatedly expressed preference for the virtual format as it reduced risk while allowing them to remain in touch with their usual psychiatrist.

### Creation of “Cohorting” or Quarantine Space

In line with international recommendations,^7,12^ consideration was given for need to “cohort” or stream new admissions according to risk of having respiratory illness in an attempt to ensure that new admissions from the community would not be bringing COVID-19 into the inpatient settings. Together with provincial partners, we liaised with detention centers to ensure that potential admissions were screened for respiratory symptoms and/or COVID status prior to coming to hospital. The hospital established a cohorting process for all other admissions to allow for a 14-day period before they transferred from acute units to other units, including for forensic patients requiring readmission from the community.

### Establishment of an Isolation Unit

International recommendations^7,12^ described the need for isolation units for large mental health institutions. The major purpose of these units is to provide a therapeutic space for persons recovering from COVID-19 with other patients who have similar infection status. This allows medical resources to be focused on supporting their recovery but does not replace medical hospitalization if needed. All patients are COVID+ in the isolation unit; there is less restriction of movement than in nonisolation units. Further, it decreases the risk of spreading COVID-19 to other patients and staff on the nonisolation unit. Infection control policies require that only COVID+ patients are cared for in the isolation unit; persons under investigation must remain under droplet and contact precautions in their home unit.

There is little guidance as to the capacity and design of such units. Given the size of our program, we established a 6-bed isolation unit within a wing of a medium secure unit with 4 bedrooms and 2 seclusion rooms. Extensive consultation with infection control was required to ensure safe separation of the isolation unit from the surrounding unit. We needed to transfer 3 patients out of existing rooms, arranged for seclusion to be provided by an adjacent unit, and made minor physical changes to the space.

The isolation unit has an open area with 2 patient beds closest to a glassed nursing station, 2 individual single rooms, and 2 seclusion rooms that could either be left open or locked according to the degree of behavioral disturbance of the patient. The unit is staffed by 3 nurses per shift, with a designated hospitalist and psychiatric attending physician. Staff rostered to this unit were not part of other inpatient clinical work, except the hospitalist. Full use of PPE was prescribed. Algorithms for medical care and criteria for transfer to a general hospital were established.

As the isolation unit was for patients who were COVID+ only, there was no restriction of movement for the patients within this unit. Priority of admission was made for patients who could not self-isolate in their home units. Patients could then return to the unit of origin following 2 negative swabs.

### Outbreak Management

The first concern about COVID+ status within the forensic program was on March 23 when a staff member became unwell on a 20-bed medium secure unit. A positive result for COVID-19 was returned on March 26, and 2 psychiatrists and 13 staff members were sent home for 2 weeks isolation. Three patients were placed on contact and droplet precautions. None of these persons became COVID+.

On March 25, 3 patients of another medium secure unit developed symptoms suggestive of COVID-19 and returned positive swabs 2 days later. This unit has 16 beds and is divided into a 9-bed area for high needs men and a 7-bed area for women requiring medium secure rehabilitation. A small number of patients are severely unwell and require long-term treatment including seclusion and can require physical restraint for day-to-day care.

A COVID-19 outbreak was declared following the COVID+ results; staff commenced wearing full PPE and patients were all placed on droplet and contact precautions. Four staff members from that unit also developed symptoms later confirmed to be COVID-19. New patient cases emerged on March 31, April 1, April 7 (× 2), April 8, and April 10 (the last asymptomatic). Of the 16 patients in that unit, 9 became COVID+.

On a second medium secure unit, a 28-bed acute admitting unit, a patient became symptomatic on March 30 and returned a positive COVID-19 test on April 1. The second patient developed symptoms on April 5 and was found positive on April 8, resulting in the declaration of a COVID-19 outbreak on that unit also. No staff members of this unit tested positive for COVID-19. With both of these outbreak declarations, the program was closed for acute admissions.

Permission was granted by Public Health authorities on April 10 to test all staff and patients on these 2 units for COVID-19 to attempt to identify asymptomatic carriage. We identified 1 patient from each of the outbreak units as COVID+. One staff member was also found to be COVID+ and asymptomatic.

The response to the outbreaks was 2-fold. On the 16-bed unit, over half the patients were COVID+ so steps were undertaken to divide the unit into 2 separate areas and stream the patients according to COVID status.

For the 28-bed acute unit, a decision was made to transfer all COVID+ patients to the isolation unit. On April 10, we transferred 2 positive patients from the acute admitting unit to the isolation unit, following up 2 d later with the transfer of the patient who was identified as an asymptomatic carrier. On April 17, a further patient who had been COVID− on April 10 developed symptoms and tested positive and was also transferred to the isolation unit. In total, 5 patients from this unit became COVID+, the last on 19 April. Our objective was to reduce the risk of spread in the larger unit and stream the smaller unit to decrease risk to the minority of COVID− patients.

In terms of severity of illness, 1 patient required transfer to a general hospital and died on April 27 after being in intensive care for 3 wk. The 12 other patients have had a mild course manageable without transfer. The first patient was declared COVID negative on April 22 and by May 7, all have become COVID negative. No further staff members have become positive in any part of the forensic program. Outbreaks were declared over on the smaller unit on May 5 and the second on May 7.

## Discussion

The COVID-19 pandemic has presented a profound challenge to health systems. The tragic deaths of patients in high-risk groups and deaths of staff members have been documented internationally. Forensic mental health services provide secure care where the transfer of respiratory pathogens is facilitated by the enclosed nature of the facilities, the high level of staffing within them, and the particular vulnerabilities and behaviors of the patient group. Depending on the facility design, achievement of social distancing may be very difficult.

Although pandemic plans and international agencies provide guidance for the need for isolation function or units within such facilities, there is no published guidance or experience regarding such units. In this article, we have described the range of interventions taken to manage the threat of COVID-19 including the establishment of an isolation unit to deal with an outbreak.

Before we had the isolation unit established and before the widespread use of PPE, we experienced outbreaks in 2 units, despite our efforts to decrease the risk of nosocomial infection. The patients who became unwell had very little or no movement outside the secure perimeter of the units. We employed the use of testing of the entire patient and staff population on the outbreak units as well as the isolation and testing of symptomatic patients to identify asymptomatic persons. By the time we had established the isolation unit, the outbreak was so extensive on the smaller medium secure unit that we could not transfer all affected patients from that unit. Instead, the isolation unit served a larger acute admitting unit, admitting 5 patients from that unit, 4 of whom were symptomatic patients and 1 asymptomatic. At the time of writing, 1 patient has died, 11 fully recovered, and 2 are COVID+ but now asymptomatic.

Responding to the needs of this population has been rapid and has presented challenges for staff and the patient population alike. Given the degree of fear among both staff and patients and our relative unfamiliarity with PPE, the transition to implement this initiative has not been without challenge. It has required heavy input from nurse educators, practice leaders, and managers to assist and support staff into the new role of caring for patients with a potentially fatal infectious disease. Clarity of communication, support from infection control and human resources, and timeliness of interventions are crucial in managing these hurdles. Further, legal responsibilities and approaches to managing uncooperative patients who present a risk to others as result of their infection required clear ethical consideration about the use of coercion to protect others. There has been a rapid and apparently successful adoption of virtual care with a need for widespread education for staff in the context of a rapidly changing environment of risk management. That change will be subject to evaluation. As well, the need to cohort patients effectively has required innovative changes to physical layouts and clinical procedures to ensure safety of our patients, especially when mixing genders. Prioritizing public health while being attentive to individual quality of life, care plans, and safety has been a common thread in managing patient care during the pandemic.

The development of the isolation unit and the testing of all staff and patients in outbreak units for COVID-19 are approaches that we believe will be central to the effective management of the outbreak. Indeed, we are proposing that should 1 patient or staff member in a closed unit tests positive that it would be wise to test all patients and staff within the environment immediately. Asymptomatic carriage has been recently described as the “Achilles Heel” of public health responses to COVID-19;^13^ testing all persons in the outbreak units yielded 3 asymptomatic carriers.

Our 100 beds in minimum secure units include units of up to 30 patients have presented challenges in controlling respiratory illness outbreaks previously are of major concern. Essential tools for future outbreak management will be rapid case identification, testing for asymptomatic carriers, and rapid movement of patients to isolation unit placement.

We have effectively managed a COVID-19 outbreak and defined some essential elements in outbreak management. Still, little is known about the acceptability of these interventions or about the impact on our patients’ mental health and staff well-being. There is a need for urgent sharing of experience and evaluation of different models of approach to outbreak management within forensic mental health services to refine and evaluate these approaches.
